# Factors Influencing the Participation of Black Older Adults in Clinical Trials on Dementia

**DOI:** 10.1093/geroni/igaf122.4237

**Published:** 2025-12-31

**Authors:** Elena Portacolone, Julio Rojas-Martinez, Kim Scott, Thi Tran, Catherine Waters

**Affiliations:** University of California, San Francisco, San Francisco, California, United States; University of California, San Francisco, San Francisco, California, United States; Bay Area Black Nurses Association, San Francisco, California, United States; University of California, San Francisco, San Francisco, California, United States; University of California, San Francisco, San Francisco, California, United States

## Abstract

**Background:**

Black communities are twice as likely as Whites to have dementia and represent 13% of the US population, constituting only 5% of participants in clinical trials on dementia. The goal of this study was to identify barriers and facilitators to participating in the a clinical trial on dementia from the perspective of Black older adults who agreed to be referred to the trialists who were testing a drug to treat Alzheimer’s disease. Between 5/1/2023-1/31/2024, we raised awareness of Black communities about the option of being referred by our team to the trial via events endorsed by the National Black Nursing Association and the Alzheimer’s Association.

**Methods:**

Interviews of Black older adults who agreed to be referred to the clinical trial identified barriers/facilitators to enroll in the trial after allowing our team to forward their details to the trialists. Interview transcripts were analyzed in ATLAS-ti with inductive/deductive content analysis.

**Results:**

In April-May 2024, a Black researcher contacted the 43 older adults who agreed to be referred to the trial. She interviewed 17 study participants who self-reported as Black/African American (average age 69, 70% new to research).

**Facilitators included:**

wanting medications, understanding the importance of participating in clinical trials, interacting with race-concordant researchers, personal exposure to dementia.

**Barriers included:**

limited connections with clinical trialists, logistical constraints, limited trust and understanding of participating in clinical trials.

**Conclusions:**

Findings underscore the importance of strong connections between clinical trialists and Black communities, as well as the salience of interdisciplinary partnerships to achieve social justice.

